# Mastitis Modifies the Biogenic Amines Profile in Human Milk, with Significant Changes in the Presence of Histamine, Putrescine and Spermine

**DOI:** 10.1371/journal.pone.0162426

**Published:** 2016-09-01

**Authors:** Marta Perez, Victor Ladero, Begoña Redruello, Beatriz del Rio, Leonides Fernandez, Juan Miguel Rodriguez, Mª Cruz Martín, María Fernandez, Miguel A. Alvarez

**Affiliations:** 1 Instituto de Productos Lácteos de Asturias, IPLA-CSIC, Paseo Río Linares s/n 33300, Villaviciosa, Spain; 2 Departamento de Nutrición, Bromatología y Tecnología de los Alimentos, Universidad Complutense de Madrid, Ciudad Universitaria, Avda. Puerta de Hierro, Madrid, 28040, Spain; Universite du Quebec a Trois-Rivieres, CANADA

## Abstract

Biogenic amines (BAs) are low molecular weight nitrogenous organic compounds with different biological activities. Putrescine, spermidine and spermine are essential for the development of the gut and immune system of newborns, and are all found in human milk. Little is known, however, about the role of histamine, tyramine or cadaverine in breast milk. Nor is it known whether mastitis alters the BA composition of milk. The BA profile of human milk, and the influence of mastitis on BA concentrations, were therefore investigated. Putrescine, spermidine and spermine were the main BAs detected. In mastitis-affected milk, the concentrations of putrescine, spermine and histamine were higher.

## Introduction

Breast milk is thought to be the optimum nutritional choice for newborns since it satisfies all the requirements for successful infant growth and development. Human milk contains many biologically active agents (including maternal immune cells, immunoglobulins, microRNAs, fatty acids, oligosaccharides, antimicrobial peptides, and some biogenic amines [BAs]) that provide protection against infectious disease, or which have functions in the development of the immune system [1]. It also contains bacteria [2] that may also be important in immune system development as well as in the formation of the gut microbiota.

BAs are low molecular weight organic bases which include monoamines such as tyramine, diamines such as histamine and cadaverine, and polyamines (PA) such as putrescine, spermidine and spermine [3]. PA play a role in cell proliferation and are essential for cell division and a range of metabolic functions, including macromolecule synthesis and structural integrity of nucleic acids. Due to its polycationic nature, polyamines can bind negatively charged molecules such as nucleic acids, proteins or phospholipids. Many cellular processes varying from gene expression modulation to regulation of enzymatic activity, and the modification of the fluidity and permeability of the biological membranes depend on such interactions. Due to this important physiological role, the PA content of cells is finely regulated by biosynthesis, degradation, uptake and excretion. PA from food significantly contribute to the total PA pool in the body and the dietary intake of PA exerts various direct and indirect trophic effects on the immature intestine, thus contributing to gut maturation. [4,5]. Despite their important physiological functions, BAs can pose health threats: consuming food in which they have accumulated (via the metabolic activity of certain microorganisms) may elicit toxic reactions [6]. In fermented foods in particular, these compounds can reach high concentrations.

Although human breast milk may be the primary source of BAs for newborns, little is known about its content in these compounds. The scant information that is available relates only to PAs, but indicates human milk to contain substantial amounts of spermidine and spermine, and lower concentrations of putrescine [7]. PA concentrations are influenced by a number of factors including the mother’s age, her genetic background, ethnicity, nutritional status, diet, how long she has been lactating, and whether her child was born at term or was premature [8,9,10]. In infants, putrescine, spermidine and spermine are essential in cell growth and proliferation, particularly in the gut [11,12].

Mastitis, an inflammation of the mammary gland, affects up to 33% of lactating women and is the main cause of unwanted early weaning. It involves the increased presence in the milk of certain species of staphylococci, streptococci and/or corynebacteria [13]. However, little information exists about the changes this may induce in the biochemical composition of human milk. In contrast, it is well known that, in bovine mastitis, the metabolism of the infecting bacteria changes the volatile compound profile [14].

The aim of the present work was to examine the concentrations of the most important dietary BAs in human milk, and to compare the BA compositions of that from healthy women and those with mastitis.

## Materials and Methods

### Milk samples

Milk samples were obtained from 40 healthy women, and from a further 30 with mastitis. The nipple and areola were first cleaned with soap and sterile water, and then with chlorhexidine. Milk samples were then collected in sterile tubes, applying manual pressure (wearing sterile-gloves) to the breast. The first drops (approximately 250 μL) were discarded to avoid chlorhexidine contamination.

### Ethical statements

All volunteers gave written informed consent to the protocol (reference 10/017E) which had been approved by the Ethical Committee of Clinical Research of Hospital Clinica San Carlos (Madrid, Spain).

### Extraction of biogenic amines

BAs were extracted from the milk samples by acid treatment [15]. Briefly, 500 μL of milk were precipitated with 100 μL of trichloroacetic acid (12% v/v) for 1 h. After centrifugation (12,500 ×*g*, 30 min) the supernatant was recovered for 1 h at 4500 ×*g* and passed through Amicon Ultra-0.5 filters (Millipore, Darmstadt, Germany). The filtrate was maintained at –20°C until analysis.

### Detection of biogenic amines

BA concentrations in milk samples were determined by ultra high performance liquid chromatography (UHPLC). For the detection of putrescine, spermidine and spermine, derivatization reactions were performed using the AccQ-Tag^TM^ Ultra Derivatization Kit (Waters, Barcelona, Spain), as indicated by the manufacturer with slight modifications. To maintain a pH of 8–10 (required for successful derivatization), 5N NaOH was added. Putrescine dihydrochloride (Sigma, Madrid, Spain), spermidine (Acros Organics, Geel, Belgium) and spermine (Sigma) standards were prepared in Milli-Q water, following the protocol indicated by Fiechter et al., [16]. The chromatographic conditions employed were those previously described [17]. Separation and the detection of fluorescence were performed using an UPLC® H-Class (Waters, Milford, MA, USA) coupled to a FLR module (Waters) (excitation and emission wavelengths 266 nm and 473 nm respectively).

Tyramine, histamine and cadaverine were determined following the derivatization, separation and quantification protocol of Redruello et al., [17]. Data were acquired and analyzed using Empower 2 software (Waters).

All the chemicals used were of the highest available purity. Water of Milli-Q quality (Millipore, Bedford, MA, USA) was used in all solutions. HPLC-grade acetonitrile (VWR, Barcelona, Spain) and methanol (Merck, Darmstadt, Germany) were used as pure solvents.

### Statistical analysis

The Chi-squared or Fisher test was used as required to examine the differences in the distribution of BA frequencies between the healthy-breast milk and mastitis-affected milk samples. BA concentrations were represented by means or medians, the associated 95% confidence interval (95% CI) or interquartile range (IQR), and maximum and minimum values (box and whiskers plots). The normality of the data was examined by the Kolmogorov-Smirnov test. The non-parametric Kruskal-Wallis test or one-way ANOVA was used to compare the BA concentrations of the two types of milk. Significance was set at *p*<0.05. Spearman correlation coefficients between different BAs were also determined. Calculations were made using either SPSS v.15.0 (SPSS Inc, USA) or StatGraphics Centurion XVII v.17.0.16 (Statpoint Technologies, Inc, Virginia, USA) software.

## Results and Discussion

**Fig 1:**
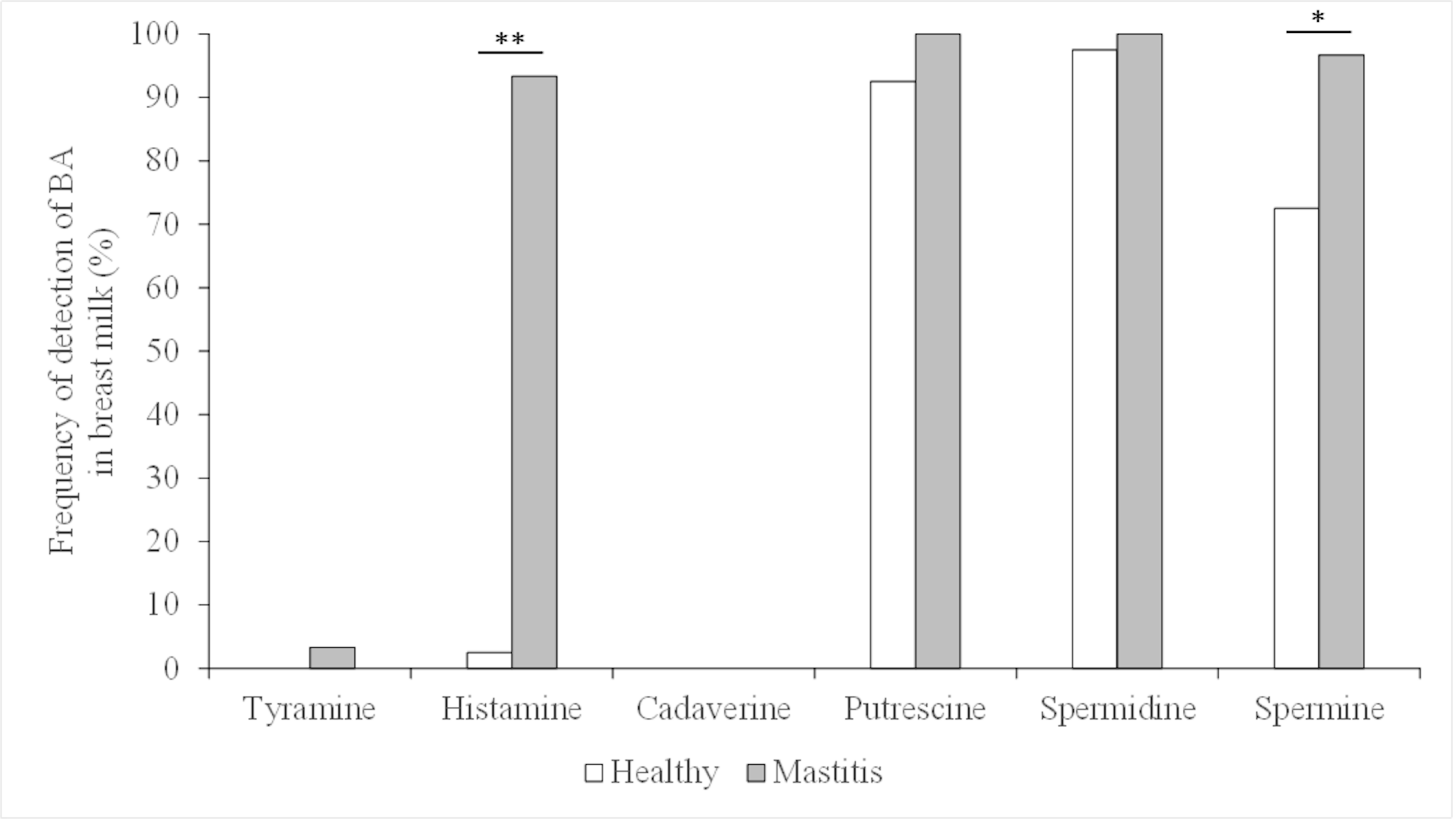
Frequency of detection of BAs in healthy-breast milk (n = 40) and mastitis-affected milk (n = 30). The results of Chi-squared tests to examine the differences in the presence of BAs between the two types of milk are shown by asterisks. *p = 0.0082; **p<0.0001.

**Fig 2:**
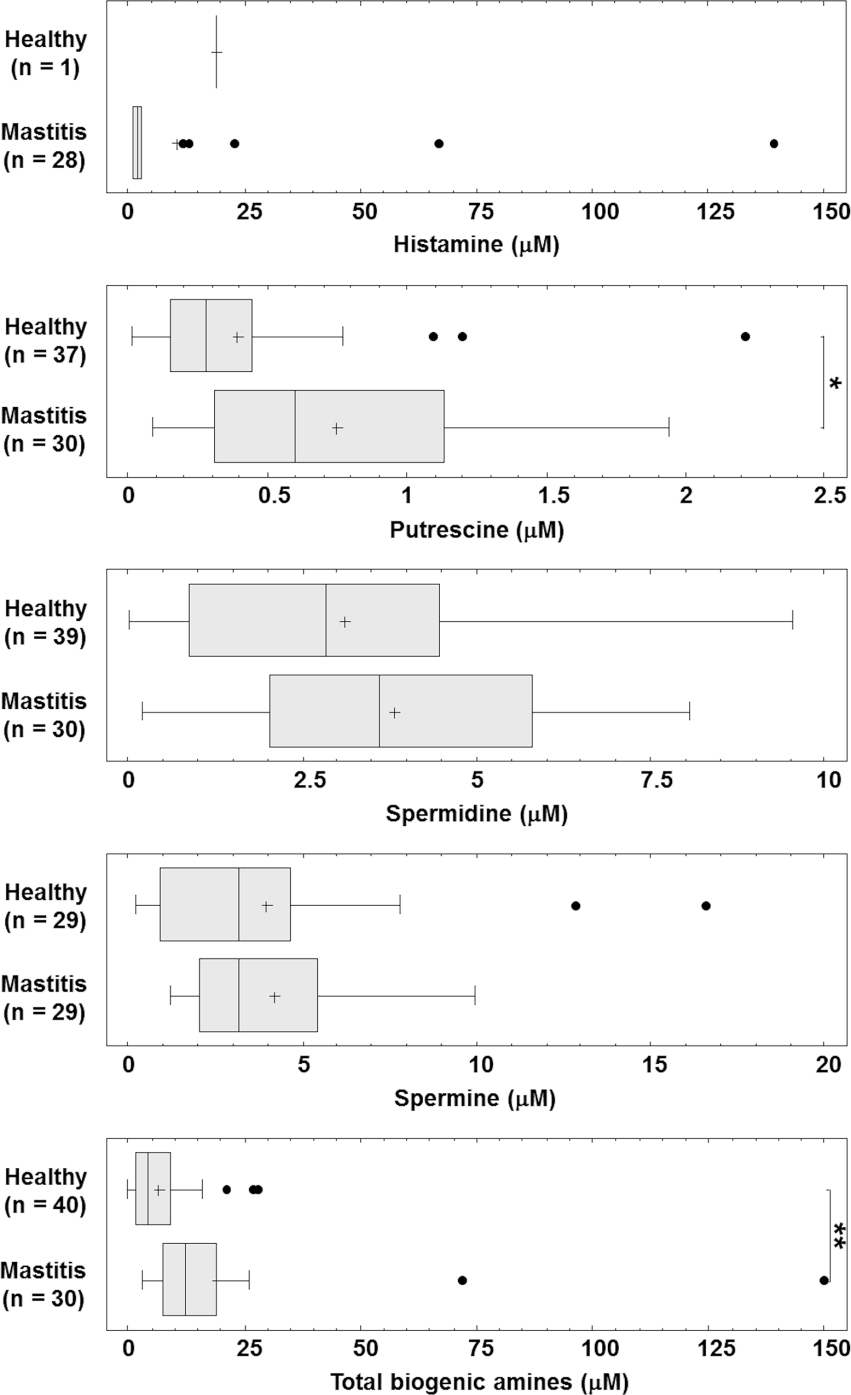
BA concentrations in healthy-breast milk and mastitis-affected milk, as determined by UHPLC. The box and whiskers plots represents means (the cross within each plot), medians (the horizontal line within the plot), interquartile ranges (total extension of the plot) and minimum and maximum values (the whiskers); outliers are represented as dots. The results of Kruskal-Wallis tests to compare the median concentration of individual biogenic amines in the two types of milk are shown by asterisks. *p = 0.002; **p<0.0001.

PAs were the main BAs found in the milk samples from the healthy mothers. Putrescine was present in 37 (92.5%) of the 40 samples analyzed, spermidine was detected in all but one sample (97.5%), and spermine was detected in 29 (72.5%) (Fig 1). Wide interindividual variation in the concentration of PAs was observed. The concentration of putrescine (0.39 [95%CI 0.28–0.50] μM) was generally lower than those of spermidine (3.12 [2.56–3.67] μM) and spermine (3.95 [3.09–4.82] μM) (Fig 2 and Table 1). This agrees with that reported by other authors [18,19]. Factors such as the length of time a mother has been lactating, and her age, are known to affect milk PA concentrations [15]. This might also explain the different PA concentrations reported in previous studies [9,18,19].

**Table 1:** Biogenic amine concentrations in milk of healthy and mastitis-suffering women quantified by UHPLC.

	Healthy (n = 40)			Mastitis (n = 30)			Comparison of	
		Median(IQR)^a^	Range(min/max)		Median(IQR)	Range(min/max)	Frequency of detection	Mean concentration
	n (%)	(μM)		n (%)	(μM)		*p* value^b^	*p* value^c^
Tyramine	0 (0)	n.d.^d^		1 (3.3)	n.d.			
Histamine	1 (2.5)	19.00		28 (93.3)	2.00 (1.00‒3.00)	1.00/139.00	<0.0001	
Cadaverine	0 (0)	n.d.		0 (0)	n.d.			
Putrescine	37 (92.5)	0.28 (0.15‒0.44)	0.02/2.21	30 (100)	0.60 (0.31‒1.13)	0.09/1.94	0.254	0.002
Spermidine	39 (97.5)	3.12 (2.56‒3.67)^e^	0.01/9.53	30 (100)	3.80 (3.17‒4.43)^e^	0.20/8.06	1	0.253^f^
Spermine	29 (72.5)	3.16 (0.93‒4.69)	0.23/16.58	29 (96.7)	3.21 (2.07‒5.44)	1.22/9.94	0.0082	0.323
Total BA	40 (100)	4.47 (1.45‒9.04)	0.01/27.74	30 (100)	4.47 (7.56‒19.03)	3.05/149.83		<0.0001

^a^ Biogenic amine concentrations are expressed as the median and the interquartile range (IQR) because data were not normally distributed, except for spermidine.

^b^ Chi square test.

^c^ Kruskal‒Wallis test.

^d^ n.d. = not detected.

^e^ Mean (95% confidence interval).

^f^ One‒way ANOVA test.

In healthy-breast milk, histamine was detected in one sample only (2.5%), at a concentration of 19 μM (Figs 1 and 2; Table 1). Tyramine and cadaverine were not detected (Fig 1). Histamine, tyramine and cadaverine are synthesized via the decarboxylation of the amino acids histidine, tyrosine and lysine respectively. It is well known that, in fermented foods, the presence of BA depends on that of BA-producing microorganisms, and on the availability of substrate amino acids [3]. The low free amino acid content of human milk may explain the absence of the above-mentioned BAs.

With respect to mastitis-affected milk, putrescine and spermidine were detected in all samples (Fig 1 and Table 1). Spermine was detected in all but one sample, a frequency significantly higher than that recorded for healthy-breast milk (97% *vs*. 72.5%; *p* = 0.008, χ^2^ test; Fig 1 and Table 1). The protective effect of spermine against inflammation may explain this difference [20].

In mastitis-affected breast milk, histamine was detected at concentrations ranging from 1 to 139 μM (media 2 [IQR 1–3] μM) (Fig 2 and Table 1), and much more frequently than in the healthy-breast samples (93% compared to 2.5%) (Fig 1). This vasoactive BA is a potent mediator of anaphylactic reactions, allergic responses, the recruitment of leukocytes following pathogen invasion, the activation of innate immune processes, and inflammatory responses [21]. It is produced and stored mainly in mast cells. As the key effector cells involved in the initial inflammatory response, these cells release histamine and other effectors when activated by infectious and other stimuli (they are not involved only in IgE-mediated allergic reactions) [21,22]. The presence of histamine in the mastitis-affected milk samples might be due to the inflammatory processes underway [23]. High histamine concentrations in milk have been also been reported in induced bovine mastitis [24,25].

Tyramine (at a concentration of 10 μM) was detected in only one sample of mastitis-affected milk. Interestingly, this same sample also had the highest histamine concentration (139 μM), the lowest spermidine concentration (0.204 μM), and was devoid of spermine (Figs 1 and 2 and Table 1). Cadaverine was detected in no sample of mastitis-affected milk (Fig 1 and Table 1).

The total milk BA concentration showed wide variation, ranging from 0.01 to 27.74 μM in healthy-breast milk, and from 3.05 to 149.83 μM in mastitis-affected milk (Fig 2 and Table 1). Mastitis is therefore associated with a significant increase in total BA (*p*<0.0001, Kruskal-Wallis test) (Fig 2 and Table 1). Although the impact on nutritional quality is negligible, the milk of women showing signs of mastitis has higher sodium, protein, IL-8 and free fatty acid concentrations, and a lower lactose concentration, than the milk of healthy women—a consequence of the opening of the mammary tight junctions [26,27,28]. These mastitis-associated changes do not, however, seem to reduce the amount of milk taken in by the infant [29].

Although differences were observed in the PA concentration of the two types of milk, their PA profiles were similar in that the concentration of putrescine was always lower than those of spermidine and spermine. The putrescine concentration of the mastitis-affected milk (0.39 [95%CI 0.28–0.50] μM) was approximately twice that of the healthy-breast milk (0.75 [95%CI 0.62–0.87] μM) (*p* = 0.002, Kruskal-Wallis test) (Fig 2 and Table 1). The spermidine concentration between the two groups of analysed samples was not significant (*p* = 0.253, one-way ANOVA) (Fig 2 and Table 1), although the concentration in the mastitis-affected milk was slightly higher (3.80 [95%CI 3.17‒4.43] μM compared to 3.12 [95%CI 2.56‒3.67] μM). A similar trend was observed for spermine, for which the concentration difference was 0.05 μM (*p* = 0.323, Kruskal-Wallis test) (Fig 2 and Table 1). DNA-PA interaction protects DNA from damage, stabilize the double helical structures and preserve its structural elasticity [5,30,31]. The binding of PA with serum proteins can enhance their stability in the blood stream and bioavailability at target tissues [5,32,33]. However, an increase in the concentration of single polyamines, is not only ineffective in DNA protection, but even detrimental, D’Agotino et al., demonstrated that 600 μM of spermine, induced the almost complete degradation of DNA [30].

**Fig 3:**
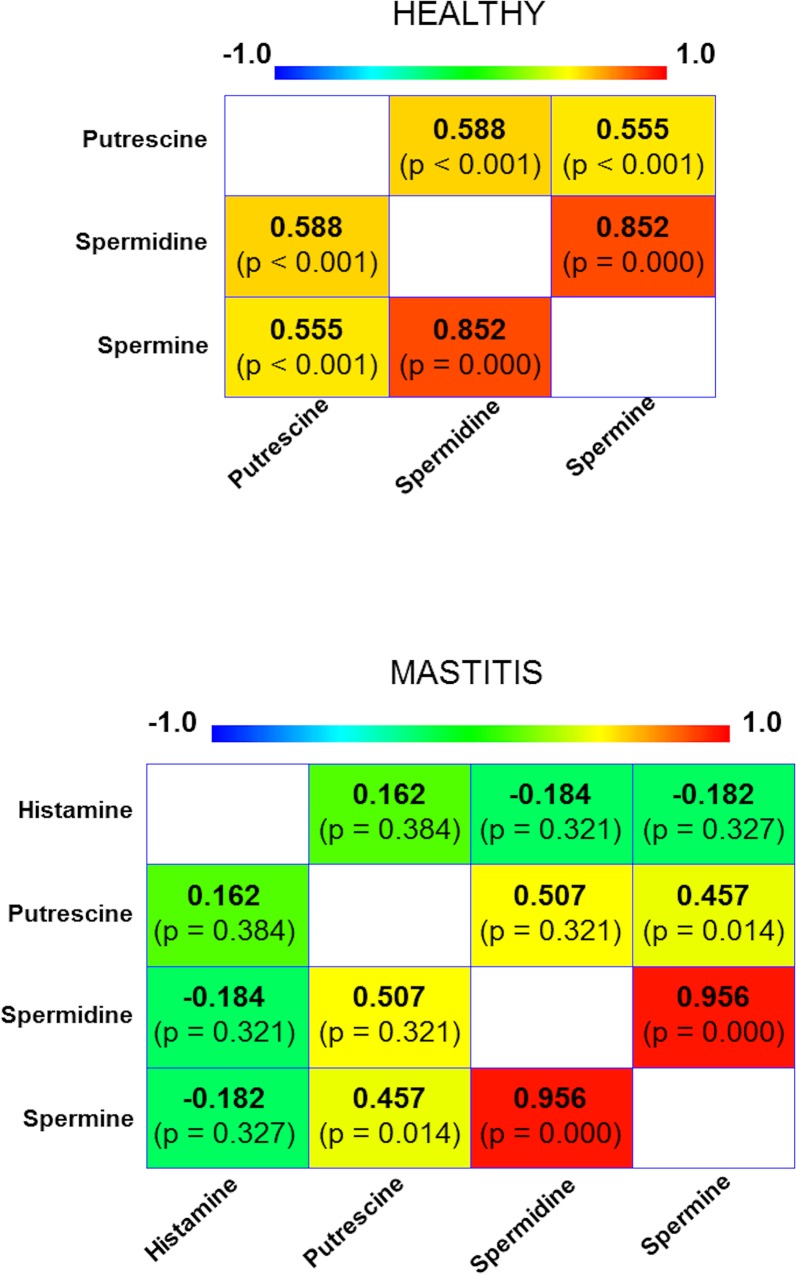
Correlation between the concentration of histamine, putrescine, spermidine and spermine in healthy-breast milk (n = 40) and mastitis-affected milk (n = 30). Numbers in bold represent the non-parametric Spearman correlation coefficient, which ranges from +1 (strong positive correlation) to -1 (strong negative correlation); the numbers in brackets show the level of significance.

No correlation could be established between the concentration of histamine and those of putrescine, spermidine or spermine (Fig 3) in mastitis-affected milk. However, the production of these PAs may respond to factors other than infection alone. Nonetheless, moderate correlations were detected between the concentrations of putrescine and spermidine (r = 0.507, *p* = 0.321), and putrescine and spermine (r = 0.457, *p* = 0.014), as well as a strong correlation between spermidine and spermine (r = 0.956, *p* = 0.000), in both types of milk (Fig 3); the close relationship in the biosynthesis of these PAs may explain these findings [20].

Some bacteria isolated from human milk have been identified as putrescine producers [34]. However, to our knowledge the contribution of the milk microbiota towards the BA content has been little examined in any mammal. It may be that the variations in the BA concentration observed in the present work are linked to mastitis-related changes in the milk microbiota [35]. The bacteria associated with the infection might contribute towards the higher BA concentrations detected in mastitis-affected milk through the synthesis of these compounds (Fig 2).

## Conclusions

To our knowledge this is the first work to report on the possible presence of tyramine and cadaverine in human breast milk, and to report mastitis-affected milk to contain higher concentrations of histamine, spermine and putrescine.

Putrescine, spermidine and spermine were the main BAs present in the milk of healthy human mothers. Mastitis-affected milk more commonly contained histamine and spermine, and had a higher putrescine concentration. Further studies are required to determine whether these differences in the BA pool may be used as indicators of mastitis and thus of possible problems in infant development.
